# Visitor Capacity Considering Social Distancing in Urban Parks with Agent-Based Modeling

**DOI:** 10.3390/ijerph18136720

**Published:** 2021-06-22

**Authors:** Zhi Yue, Jon Bryan Burley, Zhouxiao Cui, Houping Lei, Jing Zhou

**Affiliations:** 1 College of Landscape Architecture, Nanjing Forestry University, Nanjing 210037, China; cuizhouxiao@njfu.edu.cn (Z.C.); hoping324@163.com (H.L.); 2 School of Planning, Design, and Construction, Michigan State University, East Lansing, MI 48864, USA; burleyj@msu.edu (J.B.B.); zhouji10@msu.edu (J.Z.)

**Keywords:** landscape architecture, public health, risk assessment, environmental science and engineering, epidemiology, social distance, COVID-19

## Abstract

The COVID-19 pandemic has greatly influenced society in the past few years. Park accessibility and social distancing are considered important under the threat of a long-term epidemic. However, measures that can maintain park accessibility and diminish virus spreading synchronously have been seldom studied before, which may threaten public health in all major urban parks globally. This paper proposed a methodology based on an agent-based model to analyze capacities for parks by simulating park visitor behaviors when they all are social distancing. The model was derived from historical visitor data and realistic visitor behaviors in three park settings. Then, park capacities of varied contact conditions, different park policies, and layout adjustments were analyzed. First, congestions caused by social distancing without proper visitor control are found inside all parks. Second, 85 to 3972 square meters per person is predicted as a safe space in different parks. Third, the current results can be easily adjusted according to various concerns regarding infection distance and rate. Finally, it can be inferred that information provisions are more effective than space design adjustments and mandatory measures. The results can guide park managers and those who plan and design park settings. They are also helpful in improving knowledge of the mechanisms behind visitor behaviors. Moreover, these findings can be tested and verified in a variety of public spaces with many other contact-based illnesses.

## 1. Introduction

Since 2019, there have been more than 167 million confirmed infections and 3 million deaths to 25 May 2021 caused by COVID-19 outbreaks globally [1]. The high R rate (the R rate is the average number of secondary infections produced by a single infected person) [2], short epidemic doubling time [3,4], and unusual symptoms [5] made the virus extremely contagious. The constant outbreaks in different regions and potential virus mutation which may escape from the vaccine [6] make the world under a consistent threat of epidemic.

Urban parks, as a kind of public space, can be both a potential virus transmission zone and the keystone of general public health. Urban parks were closed during the strict quarantine in China [7,8], and they were partially inaccessible due to shelter-in-place orders in the USA. Meanwhile, some researchers believed that parks are important for physical activities, which will help people fight against COVID-19 [9]. Thus, restrictions to urban parks might have negatively affected vulnerable populations. However, under the continuous threats of COVID-19 [10], long-term quarantine that includes park closure may be impractical, since the benefits of physical activity in parks for both mental and physical health is well established. Meanwhile, crowds inside urban parks can be dangerous when the virus is still present in society. In China, there were 18,038 parks and hundreds of parks in and around metropolitan areas to 2019 [11]. The potential public health problems of urban parks are urgent in a pandemic background. Therefore, it is essential to understand the dynamics of behaviors in park-like settings.

### 1.1. Social Distancing as a Precaution

To diminish the risk of virus spreading in parks, some simple precautions from the World Health Organization (WHO), such as physical distancing, mask wearing, crowds avoiding, and cleaning hands, were adopted [12]. Some studies have suggested that structured schedules, a sign-up system, and social distancing [9] may be helpful. Many Chinese parks applied visitor control policies after quarantine. Most measures are intended to keep more space between people. Although the 2 m distance has been found effective under certain outdoor conditions [13], maintaining adequate individual space may be more complex. Whether the safe individual space can be calculated as a 2 m radius circle remains fuzzy. The big challenge is that the proper visitor density can be significantly influenced by park layout. Social distancing cannot be implemented without research concerning capacity control, visitor behavior observation, and behavior modeling in different park environments under a pandemic background, which have been little investigated so far.

### 1.2. Achievements of Visitor Capacity Research

Visitor carrying capacity has long been an issue in park management as one important measure controlling visitor density. The initial research of Wagar [14] developed the first formal exploration of the recreational carrying capacity concept. Since the early study on visitor impact and visiting experience in Arches National Park [15], frameworks such as the limits of acceptable change (LAC) [16], Visitor Impact Management (VIM) [17], and Visitor Experience and Resource Protection (VERP) [18] have evolved to include new factors from ecological, social, economic, and psychological research. Indicators, such as the impact of trampling [19], water demand [20], visitor use of protected areas [21], visitor-created informal trails [22], off-trail usage [23], trail degradation [24], mandatory alternative transportation systems [25], impacts of tourism hotspots [26], effects of crowding [27,28], environmental characteristics [29], ecological footprint [30], seasonality [31], community capacity [32], social marketing [33], overseas tourists [34], and visitor sociodemographic [35] have been examined. Various data sources, such as phone GPS [36], behavior observations, trail monitoring, animal habitats, soil, space syntax [37], geotagged photographs [38], and web evaluation [39] have been analyzed. The conflicts between natural resource conservation and recreational experience have been consistently reconsidered. However, the park-carrying capacity during the epidemical period and contagiousness in natural landscapes were seldom investigated [40]. Therefore, how to implement social distancing measures in parks cannot directly benefit from previous studies.

### 1.3. Patterns of Park Visitor Behaviors

The infected visitor movements and activities can be accompanied with potential virus spreading. Thus, visitor behavior patterns of gathering and moving are important factors of virus spreading. Currently, four factors have been studied: target preference, target visiting schedule, path preference, and crowds avoiding. The first factor is target preference. It has been widely found that park visitors with different intentions preferred certain destinations. For instance, popular scenery is more likely to be visited by tourists from other cities [41], trails and the main path are more frequently used by local elders [42,43], and children and youth tend to use more facilities or open spaces [44,45]. In addition, all groups will participate in landscape sightseeing [46,47]. Some other preferences, such as that of green infrastructure and architectural viewing and admiration require additional study [48,49]. The second factor is visiting schedule or staying time. This can be very different among parks and cities. Reports indicated a wide range of 1–4 h visiting time is common in Chinese parks [46,47,50,51,52,53], while earlier surveys in Nanjing indicated 1 h total visiting time and 10 to 30 min staying time at one target [47]. A trip plan of 60 min one-way isochrones was found common in western parks [54]. The third factor is path preference. Park users were found to follow other crowds as a flock of birds and use certain main paths; when they traverse off-trail, they maintain a certain radius to the trails [23,47,55,56]. Crowds avoiding is the fourth factor. Overcrowding makes visitors uncomfortable and disperse further [23,29,31,57,58,59,60]. Crowding tolerance found in Chinese urban parks is one person per square meter [61], which is much lower than that in many American parks. In conclusion, existing research indicated some park user patterns of target preference, schedules, routes preference, and crowds avoiding. Nevertheless, whether these behavior patterns are different under a COVID-19 pandemic background is still inexplicit. With insufficient reports about infections in open green spaces, we still know little about visitor behaviors in parks when considering social distance measures.

### 1.4. Visitor Behavior Modeling and Simulation

Under the circumstance that we are lacking sufficient COVID-19 cases for conducting on-site observational studies, a simulation method is adopted to study the visitor capacity and potential infection within a recreational park-like setting. The visitor behavior modeling in parks has a long history, which can be traced back to the early analysis of carriage road use in Acadia National Park. In this early framework, only empirical inputs such as travel routes and speed were involved [62]. Simulations of campsite crowding [63], probabilistic travel modeling [64], boat traffic [65], and hiker movements [66] were performed along with on-site observations. Most existing simulation models applied agent-based modeling (ABM) techniques which is widely used in the analysis of a complex system. It is found that in a complex system, the macroscopic rules of the system can be totally different from that of micro individual reactions, which is called “emergence” [67]. To understand and observe the emergent effects, stochastic models were built from the bottom up with individual agents assigned with certain attributes. An individual that is capable of ongoing self-behavior and replication is called an agent. Moreover, these agents can interact with others or the environment. The ABM differs from traditional, regression-based methods in that it allows for the exploration of the feedback loops. It can also be used as experiments that may be impossible or unethical with other techniques [68]. Early attempts were to make self-operation machines, “automatons”, eventually leading to self-playing music, robots, and clocks [69]. Such approaches led to studies of crystal formation and computer science [56,70,71,72]. Similar concepts were investigated in biology, economics, and machine learning addressing complex adaptive systems [73]. One of the efforts led to NetLogo, which is a widely used ABM software [74,75]. It is particularly well suited for modeling complex systems that develop over time, and it has been widely employed in studies of economics, biology, physics, chemistry, psychology, system dynamics, and social behavior. In the software, there are two basic agents, patches and turtles. The turtles can move and interact with patches, while the patches are immoveable. In park simulations, turtles are usually defined as visitors while patches are defined as environments. However, most previous visitor behavior simulations in parks simplified road networks into several main routes without spatial width. Then, park visitors were redistributed with probability calculated by Markov models or from observations [76,77]. The ignored elements, such as road width or joint space and the micro actions inside a target are all indispensable in models considering social distancing measures. The current simulation models must be modified to fit in new analysis.

The existing park capacity research, behavior observations, and simulations are not sufficient to support the extensive study of maintaining safe social distancing in parks. Thus, this paper proposes a simulation method with no size simplification based on ABM to study potential capacity problems in urban parks accounting for social distancing rules. The objectives of this study are listed as three essential questions as follows:

1. What is the possible carrying capacity in parks when all visitors are considering social distancing measures?
2. Are there any general problems in existing park layouts, and how will this affect the capacity?
3. What are the possible policies and design adjustments to support social distancing measures in current parks?

## 2. Materials and Methods

**Figure A1:**
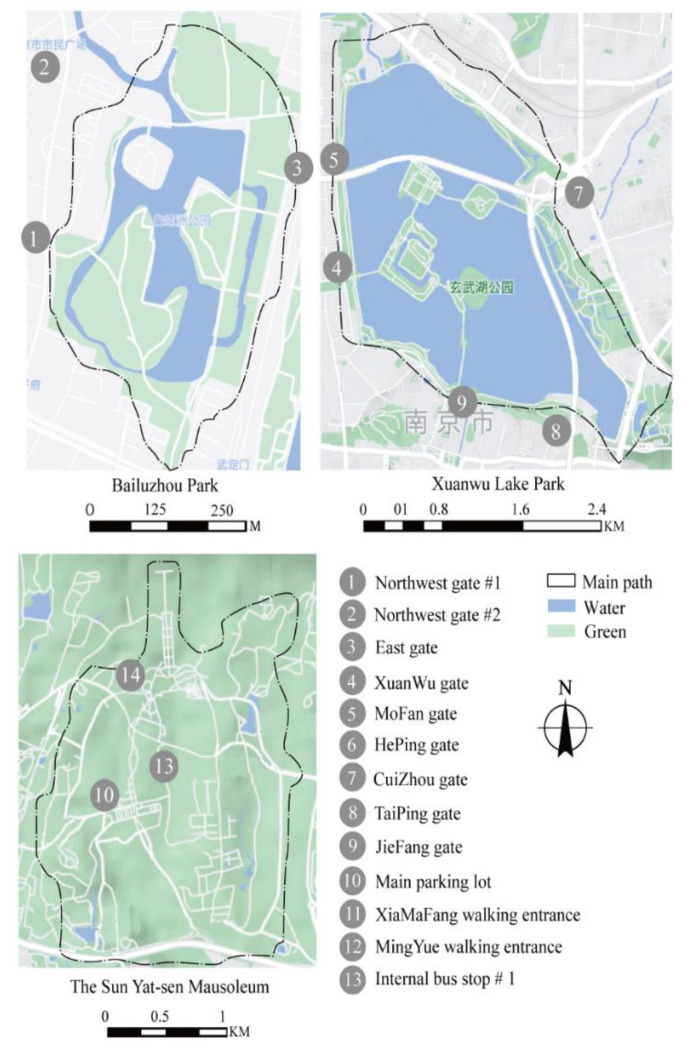
Topography of three parks. The base map is acquired from Google Maps. Copyright 2020 © Author, all rights reserved, used by permission.

**Figure A2:**
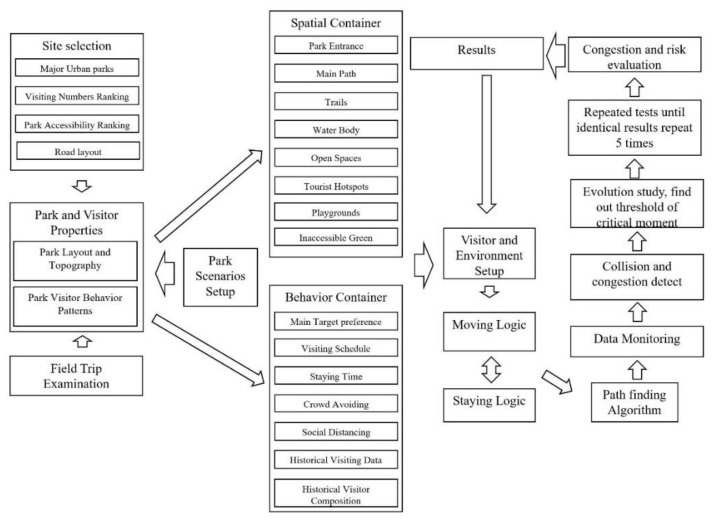
The diagram of workflow. Copyright 2020 © Author, all rights reserved, used by permission.

The research is aimed at revealing potential capacity problems in parks when all visitors are considering social distancing measures. It includes five steps to build and apply the ABM as follows:

First, the three most visited urban parks with different typical layouts in downtown Nanjing were selected (The topography of parks are shown in Appendix C Figure A1). Second, a framework of visitor behaviors that includes maintaining social distancing was established. Third, ABM with different park environments and historical visiting data were repeatedly tested under varied infection assumptions. Fourth, the risky spots were identified and analyzed. Finally, the supporting policies and design adjustments were compared. The workflow can be found in Appendix C Figure A2.

### 2.1. Site Selection

**Figure 1:**
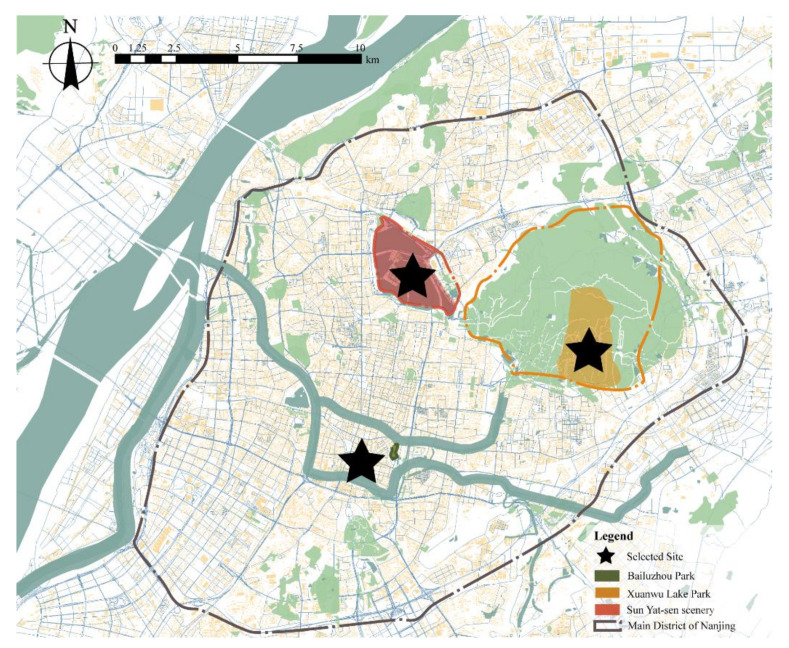
Site selection in downtown Nanjing. Copyright 2020 © Author, all rights reserved, used by permission.

Three popular parks, as shown in Figure 1, were selected based on two factors, yearly visiting numbers and road layout. They are Bailuzhou Park, Xuanwu Lake Park, and Sun Yat-sen Mausoleum Scenic Area.

**Figure 2:**
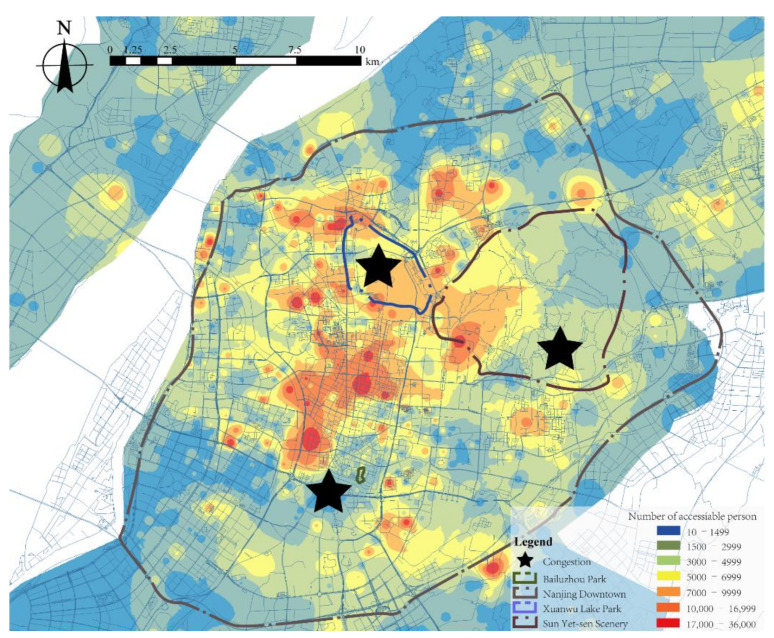
Park accessibility in downtown Nanjing. Copyright 2020 © Author, all rights reserved, used by permission.

First, nearly 46% of tourists entered these parks during the Spring Festivals and other holidays [78]. Moreover, they are easily accessible sites in downtown Nanjing, as shown in Figure 2. Thus, they are the most sensitive targets of virus transmission due to overcrowding.

**Figure 3:**
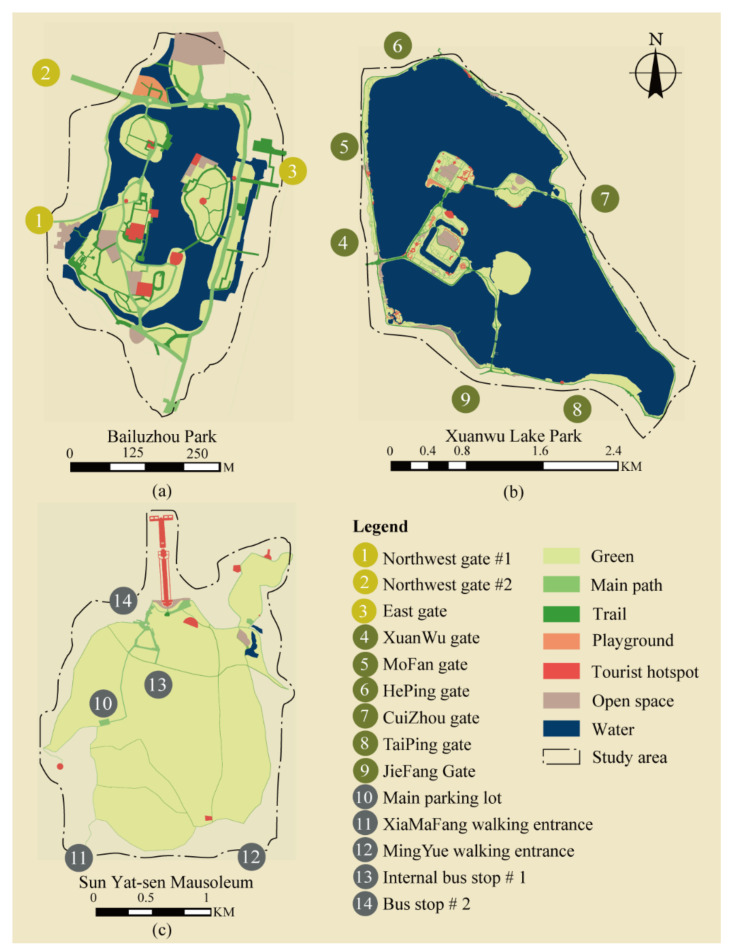
Parks Layouts. (**a**) is the layout of Bailuzhou Park; (**b**) is the layout of Xuanwu Lake Park; (**c**) is the layout of Sun Yat-sen Mausoleum. Copyright 2020 © Author, all rights reserved, used by permission.

**Figure 4:**
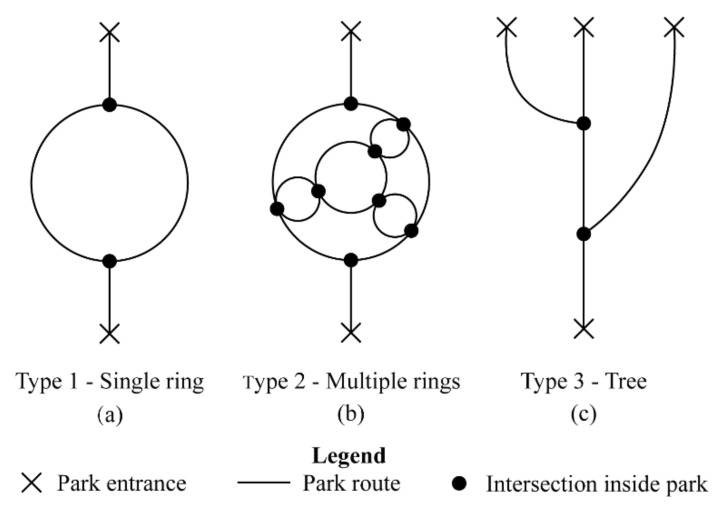
Road structure patterns of three parks. (**a**) is the single ring pattern; (**b**) is the multiple rings pattern; (**c**) is the tree pattern. Copyright 2020 © Author, all rights reserved, used by permission.

Each park represents a unique type, as shown in Figure 3 and Figure 4. The Bailuzhou Park is famous for its history as a modern park transformed from a traditional garden (Figure 3a). Nowadays, it serves as a park for nearby communities and tourists. It is the smallest, covering 15.28 ha, with a water body and several islands inside. Most tourist hotspots and open space are on these islands and connected by a circular path. There is only one open space serving as a children’s playground with facilities near the north entrance. The trail systems are near the east and south coast. The road structure can be simplified as a ring, as displayed in Figure 4a. Xuanwu Lake Park is the most famous park in downtown Nanjing as a historical lake and royal garden. It is the biggest central park for downtown Nanjing, which covers 513 ha. The main path is composed of circles within circles and connects smaller scenery spots. There are many tourists’ hotspots, open spaces, children’s playgrounds, and a long trail system inside it. The tourists’ hotspots are usually designed as a garden with long curving paths inside. The layout can be simplified as multiple rings, which is common in many big urban parks (Figure 4b). The Sun Yat-sen Mausoleum is the core of the Golden Purple Mountain scenic area and also used as an urban park. It has an area of nearly 955 ha and is famous for the culturally significant tomb, Sun Yat-sen Mausoleum (Figure 3c). The path to Sun Yat-sen Mausoleum is linear, connecting several parking areas and long trails scattered in scenic areas (Figure 4c). This is a typical layout in commemorative parks.

### 2.2. Visitor Behavior Modeling

The simulation implemented in this study is based on an ideal simplified situation, in which park visitors will maintain social distancing as their first priority. It is hard to determine what percentage of park visitors are willing to maintain social distance in reality without sufficient observation. However, this simplified assumption and corresponding results can be easily adopted in the future with suitable data.

The workflow of the modeling is shown in Appendix C Figure A2. To initiate an ABM, the master plans of parks were inspected with field observations. Targets were defined by facilities and activities. The accessible areas, which are mainly roads, open space, and open lawns (some lawn with shrubs and flowers are forbidden to access) were defined in the program. All spatial dimensions were kept to 2 m resolution. Then, behavior rules and visitor data within the setting were inputted. However, early test runs highlighted that complex visitor behaviors in the program with similar spatial resolution (6 ft or 2 m) is impractically long. This is why the study is limited to a total of five behavior rules in the simulation.

**Figure A3:**
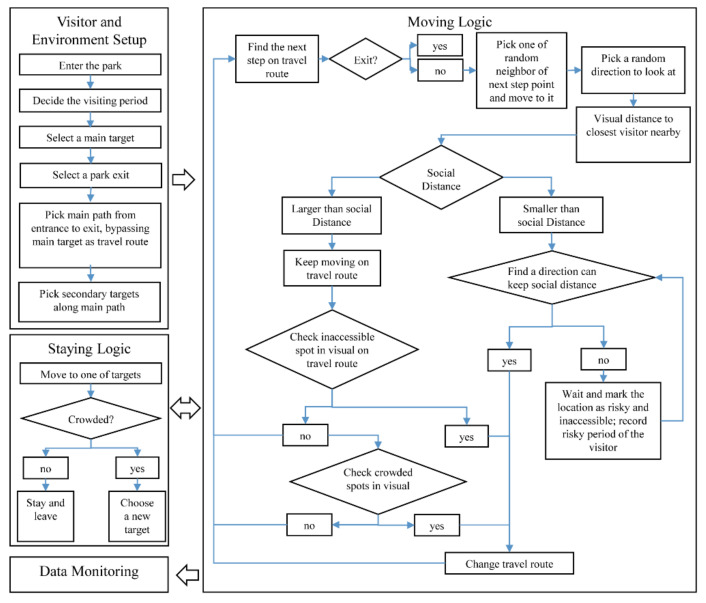
The diagram of moving and staying logic. Copyright 2020 © Author, all rights reserved, used by permission.

The first rule is to maintain social distancing. First, each visitor is programmed to maintain a minimum 2 m distance from each other when possible (the details are shown in Appendix C Figure A3). The visitor is also programmed to prevent from entering a location that is incapable of maintaining social distance. If a visitor is already at a position that fails to maintain social distance, the visitor will stop and wait for others to move away in the current study.

The second rule is making travel plans, which involves visiting preferred destinations and choosing popular paths. Each type of visitor is assigned to the preferred main targets. The types of preferred targets are simplified into four categories: popular scenery, trails, open space, and entertainment facility. Each target category has its own visitor type according to earlier surveys [47]. Tourists visit popular scenery; exercisers use the trails and the main path; leisure visitors try to locate open spaces and waterside spaces, while those with children attempt to find entertainment facilities. Sightseeing is not set as a separate target, as it cannot be distinguished from other behaviors. All destinations were examined according to the fieldwork, as shown in Figure 3. After that, the travel route from the entrance to the exit bypassing the main target is determined. Finally, secondary targets are randomly picked along the travel route. This progress can be interrupted and repeated when the visitor has to choose a new target. The number of secondary targets is limited by the staying time.

The third factor, staying time, is based on previous reports [47] and on-site surveys. The detail of the survey can be found in Appendix A. The simulated visitors are coded to spend 20 to 30 min in their prior target and 10 to 30 min randomly in other targets. The traveling time to the main target will be estimated and added to the total visiting time, which limits visitors with short visiting plans to go deep into the park.

The fourth rule is path selection; when there is more than one path to choose, the wider or more used paths were predefined as preferred ones. If a position that is congested or crowded is in a visible radius, the visitor is coded to choose another route and target unless it is the only option.

The fifth behavior is to prevent crowding. When a visitor arrives at the target spot, the visitor will judge whether there is more than one person per square meter in the visible radius. Then, the visitor will decide to stay or move on to another target.

### 2.3. Visitor Data

ABM utilizes realistic data of visitor quantity, types, and plans for better simulations. The simulation employed data at park entrances instead of the unavailable data of every spot and paths. Visitor numbers were counted by the park management and manually verified by the research group. The data of Bailuzhou Park and Xuanwu Lake Park lasted for 14 h from 6 a.m. to 8 p.m. In Sun Yat-sen Mausoleum, it lasted 12 h from 7 a.m. to 7 p.m. These inspections were conducted from 2019 to 2020, including at least two weekdays and two weekends for each park. The data of 2019 are used as the maximum limit of visiting quantity after the park reopened in 2020. The data of the most visited entrances are shown in Table 1.

The composition and plans of visitors are shown in Table 2 and Table 3. The data were acquired by questionnaires conducted at park gates and parking lots. The details of the survey instrument can be found in Appendix A. The numbers of successfully received questionnaires in Bailuzhou Park, Xuanwu Lake Park, and Sun Yat-sen Mausoleum were 343, 566, and 330, respectively. Most park visitors stayed for approximately 1 h, while those to scenic areas stayed for 2 h. During the weekends, the average length of staying was longer. Finally, the average of 10 h duration was used for all parks, excluding the periods 6–7 a.m. and 7–8 p.m.

### 2.4. Model Programming

The simulation was based on Netlogo 6.12, which is a multi-agent programmable modeling environment. The turtles in Netlogo represented the visitors, while the patches were used as environmental information. An algorithm called A-star was adopted to simulate the path-finding process. Some variables were pre-defined to review the states of the environment or the visitors, such as the main target, traveling time, overall length, and places where social distancing fails.

To simplify the relationship between visitor quantity and simulation result, some differences of visiting numbers were ignored. The hourly changes of the visitor quantities at each gate were simplified at the most popular entrances. Meanwhile, the number of daily visits at the gates was kept different.

### 2.5. Simulation Refinement and Capacity Algorithm

The simulation of Xuanwu Lake Park was tested first without the consideration of social distancing measures. The result was compared to previous observations [47] and found to be similar. Then, more tests were performed. Since the result can be unstable because of random behaviors, five out of ten identical results are required for the final confirmation.

By assuming that the spread of COVID-19 is the consequence of a failure in maintaining social distancing, the ideal capacity should be defined as the daily visitor quantity that will produce no failure. However, this rigid condition is not applicable neither in reality nor in simulation. Thus, the consequences from the simulation of the Bailuzhou Park were inspected by increasing daily visitor numbers.

**Figure 5:**
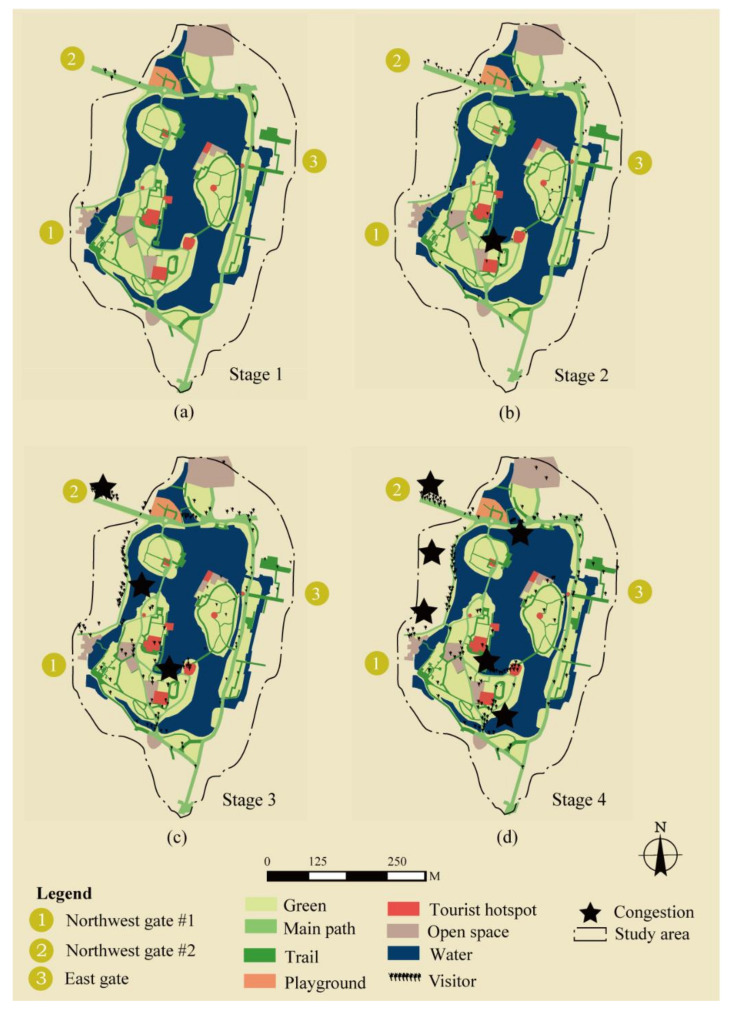
The four stages of congestion evolution in Bailuzhou Park. (**a**) is stage 1; (**b**) is stage 2; (**c**) is stage 3; (**d**) is stage 4.From (**a**–**d**), more users enter the park. There is no congestion in stage one but one congestion in stage two and more congestion in stage three until the entrance was blocked in stage four. Copyright 2020 © Author, all rights reserved, used by permission.

The evolution of results is illustrated across four stages in Figure 5. The first stage is when very few visitors entered the park; several random collisions could be observed when two visitors met on the path. After more visitors entered, some collisions clustered into congestion. When 5 to 10 visitors entered the same narrow trails without acknowledging how many visitors were already inside, it became congested later. This could be recovered after all visitors exited the trailhead and chose another path. This period is defined as stage two. If more visitors entered the park, the congestion will grow. When the congestion spread to the main path, it became unrecoverable. Site-wide traffic crowding could be observed. This is defined as stage three. The fourth stage happened when there were too many visitors at the entrance, causing it to become blocked.

The second stage is the beginning of later congestions. In addition, it is when visitors involved in the congestions began to be exposed to a continuous risk due to close contacts. By comparing different parameters, three visitors that are unable to maintain social distancing for 10 min were found to be the threshold for the second stage. This is defined as a critical period of 10 min as the beginning of potential infections. The corresponding total visitor number is defined as the park capacity considering social distancing measures. However, the outdoor dynamics of COVID-19 are still unknown. Neither the safe distance nor the critical infection span has been confirmed yet. This study also proposes a large range of capacities under different assumptions, which include different contact time spans of 1, 2, 4, 8, 16, 32, and 64 min. By comparing the results, the limitation of critical period of 10 min assumption can be improved and covers a larger range of potential impacts of social distancing in parks.

Moreover, the results of possible park management policies and park adjustments will be examined. Controlling the spread of COVID-19 can be categorized under two improvements: visitor control measures and design adjustments; three possible branches of visitor control policies and corresponding capacities were investigated. The first is when park management closes certain types of targets inside the park. Under this setting, only some visitors will enter the park for targets that are still open. The second is when park only opens for limited hours. The first period is from 7 a.m. to 11 a.m. and the visitors are mostly local physical exercisers. The second is from 11 a.m. to 3 p.m., when all types of visitors enter the park. The third is from 3 p.m. to 7 p.m., when local physical exercisers and those for social leisure enter. The period lengths are all equally set to 4 h. The third is when visitors apply different preventing strategies. The previous simulations assumed that people would stop and leave if they were unable to maintain social distancing measures in all directions, which is defined as passive mode. Another strategy is coding visitors to leave the risky spot as soon as possible, which is defined as aggressive mode. Two results of varied strategy will be compared. Meanwhile, the consequences of potential park layout improvements can be studied, which may include partial path adjustment and scenery hotspot adjustment. These studies of capacity under varied scenarios can be helpful in determine better solutions for park managements in epidemic settings.

## 3. Results

### 3.1. Carrying Capacity of Different Critical Infection Periods

As shown in Table 4, the capacities in different parks were calculated. The historical visitor quantity and coarse estimation of visitor capacity based on social distancing space requirements were used as the two important baselines. The first is the daily number of visitors before the COVID-19 breakout, which is the possible maximum limit after the parks reopen. The second is equivalent to the accessible area (the area that visitors can walk, including path, open space, open lawn; not including lawn with flowers and shrubs where visitors should not access) divided by the social distancing circle area, i.e., 12.56 m^2^ (by assuming each person keep 2 m distance from others in all directions). The simulation results displayed that each visitor acquires 85 to 3972 square meters in different settings. These are only a very small portion of either the historical data (0.5–8.9%) or the coarse estimation (0.3–14.7%), which also differ significantly among parks.

Under different critical period assumptions, all park capacities decrease with the critical infection length. However, even the best result is still lower than the historical records.

### 3.2. Congestion Points

**Figure 6:**
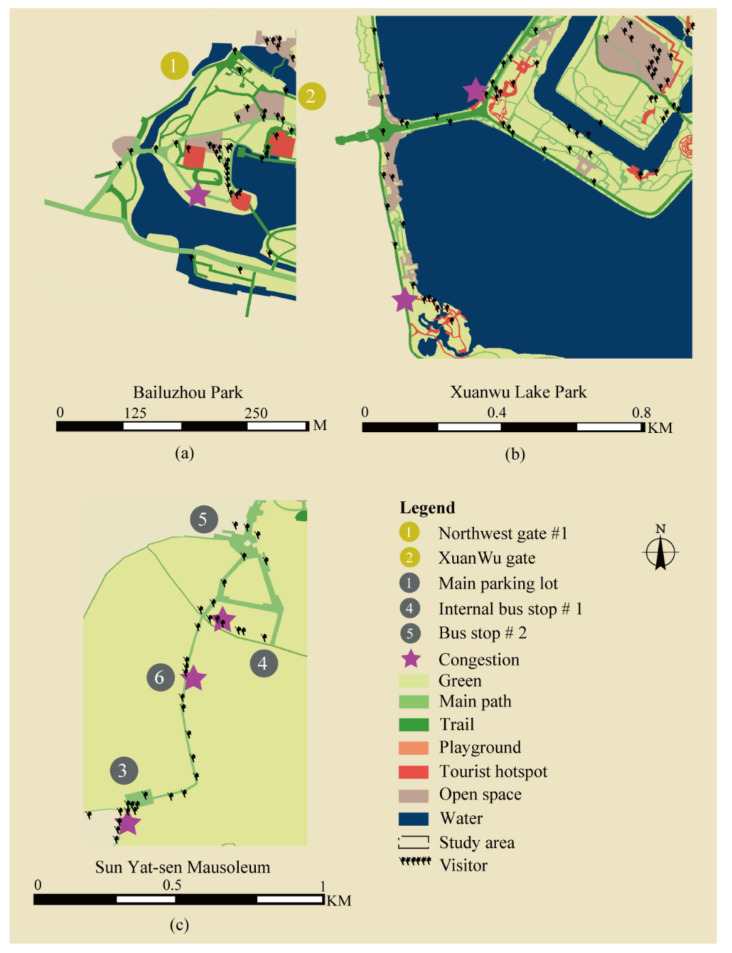
Congestion points in parks. The tiny human shape figure in maps represents a single visitor. Black stars display places of congestion. (**a**) is the map of congestion in Bailuzhou Park. The congestion is on a trail between two destinations. (**b**) is the map of congestions in Xuanwu Lake Park. The congestions are inside trails in the destinations. (**c**) is the map of congestions in Sun Yat-sen Mausoleum. The congestions are near the parking area and on the long trail to the Mausoleum. Copyright 2020 © Author, all rights reserved, used by permission.

Several typical congestion points are studied as shown in Figure 6. In the Bailuzhou Park, congestion constantly occurred in a narrow trail connecting two destinations (Figure 6a). In the Xuanwu Lake Park, there were two frequently crowding places, which are two popular sceneries with narrow trails inside (Figure 6b). In the Sun Yat-sen Mausoleum, congestion usually occurred in long narrow trails from the parking area to the Mausoleum (Figure 6c). High-density spots could also be expected on other long trails. All congestions occurred in the middle of narrow shortcuts between two visiting targets. It is possible that the visitors’ preference and unseen destinations cause congestion. In Bailuzhou Park and Xuanwu Lake Park, congestion occurred locally outside main pathways, while the main pathway in Sun Yat-sen Mausoleum was congested and caused significant park capacity reductions.

### 3.3. Capacities under Different Scenarios

Three visitor control situations were examined with the simulation of Bailuzhou Park. The first is when park management closes certain types of targets inside the park. The second is limiting the park’s opening hours. The third is advising visitors using a more aggressive avoiding strategy. The results in Bailuzhou Park are shown in Table 5. The first measure usually decreases the capacity because more visitors of the same intentions may get congested at the same targets.

The capacities of three limited opening periods were tested and compared. The results of the first (7 to 11 a.m.), second (11 a.m. to 3 p.m.), and third period (3 to 7 p.m.) are 265, 445, and 355 people, respectively. The maximum capacity is when there are all kinds of visitors entering the park.

The results of two different preventative strategies were compared, which is 380 for passive mode and 255 for aggressive mode. Although walking away quickly seems safer, it actually accumulated risky moments when passing.

The simulation displayed that the first and second policies are capable of increasing the capacity from 1.09 to 1.17 times alone, while the different preventative strategies may decrease capacity.

### 3.4. Capacities of Different Design Adjustment

The majority of the park spaces were not occupied when congestion occurred, which implied that by preventing the congestion, it may improve total park capacities. Several results of different park space modification can be tested, including park adjustments and AI measures.

**Figure 7:**
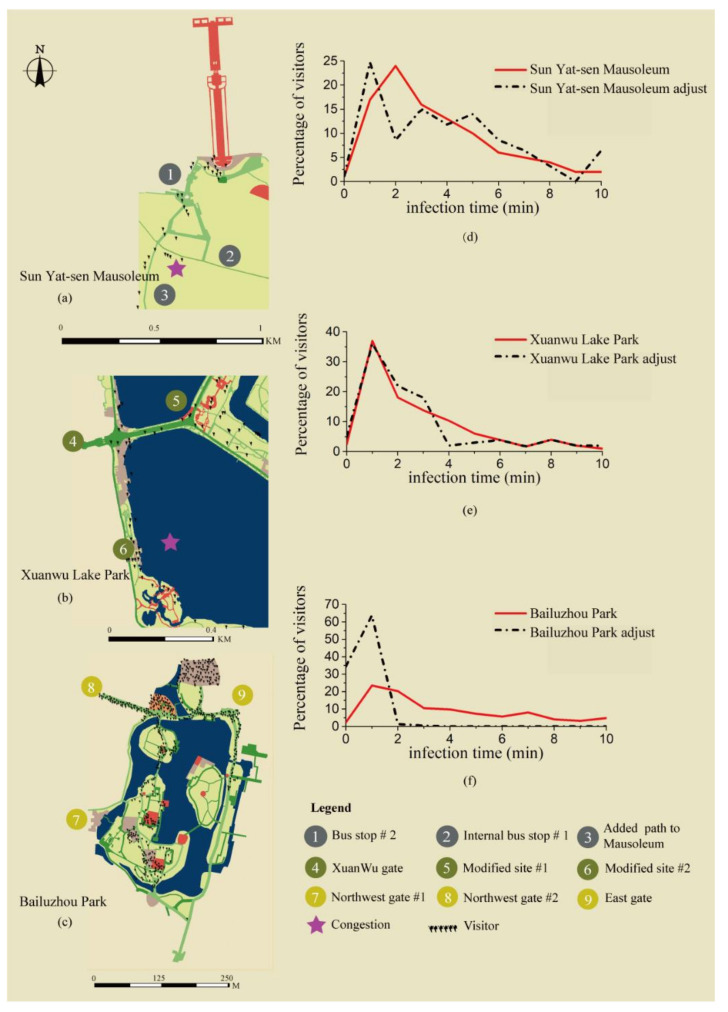
New congestion points in adjusted parks with changed contact periods of visitors. The tiny human shape figure in maps represents a single visitor. Purple stars display places of congestion. (**a**) is the visitor distribution map in adjusted Sun Yat-sen Mausoleum, which has a new congestion; (**d**) is the comparison between visitor infection time before and after park adjustment; (**b**) is the visitor distribution map in adjusted Xuanwu Lake Park, which has one congestion; (**e**) is the comparison between visitor infection time before and after park adjustment; (**c**) is the visitor distribution map in adjusted Bailuzhou Park, which has no congestion; (**f**) is the comparison between visitor infection time before and after park adjustment. Copyright 2020 © Author, all rights reserved, used by permission.

The modified Sun Yat-sen Mausoleum solution was tested by adding an additional one-way path that connects Mausoleum and the parking lot. As shown in Figure 7a, the capacity increased from 128 to 152. Moreover, visitor contacts of 2 min decreased, as shown in Figure 7d. Inspection reveals that although congestion on the main path to the parking lot disappeared, new congestion appeared on other narrow trails again. Thus, adding a partial path will not sufficiently reduce the risks of the virus for the entire scenery area.

The second adjustment is based on the correlation found in Table 5. The connection width to the main path is highly related to the park capacity. Thus, it is assumed that an increase in the connection width can improve capacity. Two congestions from the Xuanwu Lake Park were selected and modified as shown in Figure 6b. The modified models increase the opening width to a 10 m maximum. The daily capacity increased from 836 to 1206, as shown in Figure 7b. However, the increased capacity generated congestion within the target areas later. In addition, visitor contacts of 4–5 min decreased as shown in Figure 7e.

The third solution applied is based on observations of how visitors were trapped inside trails. The simulation implied that before they entered the trail, they do not know how dangerous it is inside. The AI warning boards are added at all destination entrances, giving warning signals if the visitor quantity inside is dangerous. This solution in Bailuzhou Park greatly reduced the congestion and improved the capacity to 820. In addition, the distribution of visitors inside the park became uniform in all open spaces, as shown in Figure 7c. Moreover, the percentage of visitors’ contacts longer than 2 min dramatically decreased, as shown in Figure 7f.

The above adjustments indicate that information provision at the entrance point is more important than space design alone. The greatest merit arises from the well-informed reaction of park visitors.

### 3.5. Capacities under Different Assumptions

**Figure 8:**
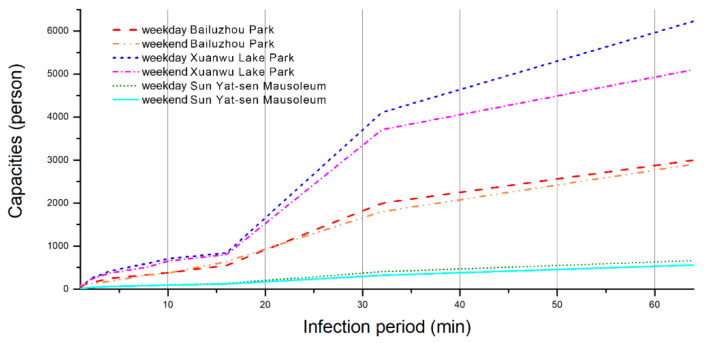
Capacities of different critical periods. The capacity usually grows as the critical infection periods arise. The results of weekday and weekends are similar. The capacity of bigger parks is usually higher, as shown in the difference between Xuanwu Lake Park and Bailuzhou Park. If the park traffic system is flawed as at Sun Yat-sen Mausoleum, its capacity will be very limited. Copyright 2020 © Author, all rights reserved, used by permission.

The changing rate of capacities under different assumptions is shown in Figure 8, and there are two significantly different segments; the first segment is from 1 to 16 min, and the second is from 16 to 64 min, where the slope of the latter is much steeper. The slopes of the first and second segments are semi-linear, which implies that the capacity increases approximately linearly with the critical infection period.

## 4. Discussion

The simulation results displayed varied park capacities in different typical parks. The visitor density can be varied from 0.0117 to 0.0003 person per square meter. It is congestion that prevented capacities from increasing. In circular layout parks, congestions happen outside the main path, while in linear layout parks, congestion happens on the main path. Several visitor control policies can improve capacities from 1.09 to 1.17 times alone. Moreover, park adjustment can improve capacities from 1.18 to 2.15 times alone. The results indicated some important information, including general risks of typical urban parks, disadvantages of the current layout, consequences of varied measures, and possible ABM-related theories. These indications and implications are discussed below.

### 4.1. Risks in Urban Parks

First, the above results show that there is a high risk of COVID-19 infections in urban parks if infected visitors enter. The capacity of simulation is much lower than that of historical data and coarse estimation based on social distancing measures. It is also much lower than the currently performing visitor control numbers, which is between one-third to one-half of historical data. The best capacity of Bailuzhou Park is only 8.9% of historical data. Even with the most optimistic assumption, the capacity is 72.8% of historical data. For other parks, the result of optimistic assumption can be as low as 3% of historical data.

Meanwhile, the best result in Bailuzhou Park is 5.4 times the average park area per person in Nanjing [79]. This not only suggests that visiting numbers should be limited in the three studied parks but also implies a general shortage of public green space in the city if social distancing measures are implemented. This shortage may become a long-term risk in all urban parks in another pandemic.

Second, there are potential flaws in park layouts. All parks were designed for gathering and relaxing, rather than for dispersing. The park layouts selected are typical in urban parks. The congestions occurred more easily in the main path of a linear layout. Meanwhile, congestion occurs more in shortcuts between destinations in circular layouts. The destination spots with long paths inside and limited openings to the outside are more easily congested. All these common structures in urban parks may be potentially dangerous destinations. These also imply that all traffic planning in current parks should be reconsidered overall. The layout should be more circular, and trails inside destinations should be shorter. The connection width to the main path should be wider. While universal modifications to all destinations can be expensive and impractical, providing density information inside to outsiders with an AI board may be a better option.

### 4.2. Emergent Effects of Visitor Behavior and Enviroment Interactions

Urban parks, as a kind of public space, are designed for entertainment and gathering. During the pandemic, they are also vulnerable spots. However, testing contagions inside a park can be hard or unethical; ABM supplies a substitution for the unrealistic experiment and gives us a chance to observe how interactions between visitors produce emergent effects in parks.

Three factors were found to be important to explain places of congestion. First, all congestions were found in a 500 m radius of the most visited entrance. Second, popular scenery with long narrow trails inside are more easily become congested. Third, the correlation between the capacity and connection to main path is significant, as found in Table 5. These can all be explained as the result of visitor behavior or keeping social distance. Each visitor acquires a 2 m radius circle to keep safe social distance, which is 12.56 m^2^. Nevertheless, two microstructural factors prevent visitors from uniform distribution. The first factor is trail width. All trails that are narrower than 2 m can only be walked in one direction safely and slowly. If the narrow trail is long or less connected to the main path, it will easily become congested. Open spaces and children’s entertainment sites are seldom congested because they are more visually open. The trail systems are less crowded because they usually have multiple exits in short distances. However, exits and entrance of the scenery spots in the parks are usually planted with trees and shrubs. This lack in providing important visual information may cause congestions later. By applying an opening entrance or providing information via an AI board, the park capacity can be greatly improved. Another factor is the distance to the park entrance. Since most visitors have a limited visiting time and pace speed, the targets near the entrance are more easily picked as targets. These properties of visiting targets greatly affected the capacity, which result in a significantly larger individual space, 85 to 3972 square meters in varied park settings. This displayed how greatly the microstructure and visitor behavior interactions can affect park capacity decisions. It also warned us that the capacity calculated without simulation can be risky and overestimated.

### 4.3. Mechanism of Results Explained with ABM Simulation

Many public health studies are highly dependent on observations. However, for timely issues, important reports may be too late. Meanwhile, it is hard to test and compare results of different public health precaution policies since it is in a complex system, which is hard to predict. In addition to working as an alternation for experiments, ABM is also a good tool for park scenario research in a pandemic, since it can be used as a tool to understand the mechanisms behind results. Three important nonlinear characters in a complex system can be found and studied.

The changing rate of capacities under different assumptions has a clear marginal decreasing effect, as shown in Figure 8. By checking visitor parameters, it is found that this is possibly affected by the total visiting time. As many visitors in the parks had a 1 h schedule, they were less likely to become infected for more than 32 min.

It is found that the results of visitor management policies globally have less benefits than partial park adjustments. Some even decrease the total capacity, such as partial closure. By examining and comparing target data between different solutions, it can be inferred that the congestions in targets were caused by visitors of similar intentions. When the number of places that can be visited is limited, more users selected the same targets, making them more risky. A globally reasonable idea can produce unexpected local problems in a complex system.

It is also found that some results of polices will be greatly changed with different initial conditions or assumptions. An AI warning board is a tiny modification. However, since it solved the information provision problems, this little change in initial conditions produced a much more uniformly distributed space pattern of park visitors. By checking and observing how simulated visitors make their decisions, it is found that they changed their targets immediately instead of creating congestion in the pathways. Consequently, more targets can be used as per usual.

It is important to note that when all the explanations are important for current public health issues in parks, they are also important footprints for understanding the complex system. ABM can be an important tool to study mechanisms of public health issues in the recent future.

### 4.4. Connections to Existing Studies

The COVID-19 pandemic could fundamentally change society’s way of life [80]. Some questions regarding public space design has already been raised [81], and short-term density reduction has been implemented in terms of tourism [82]. In addition, fewer human activities have been observed during and after the pandemic [83,84]. A previous study has shown that the timings of the social distancing policy is important with a similar ABM [85], but most findings are either related to epidemics or their side effects. The findings in this paper highlight potential design problems in parks when social distancing measures are introduced. Nevertheless, studies relating to the spread of epidemics in parks are still insufficient. As more results and data become available, one may be able to construct reliable infection rates [86,87] for these recreational areas and reliable risk analysis values [88,89].

It is widely believed that this epidemic may have profound consequences to life as it was before [88]. Mental pressures have been inspected and revealed [89]. Digital life has been encouraged; novel design and planning processes are being suggested and implemented [90,91]. It seems that a new low-density virtual lifestyle is becoming commonplace. However, the benefits of urban parks are well established, and their historical layout should be respected. Even without significant change, suitable alternations or policies may help more citizens utilize the parks as they were before at a suitable social distance, as discussed in this study. The results from this study also suggest that providing enough information is more effective than mandatory measures. This new finding should be added to the checklist of landscape architects and environmental managers. Currently, the infection control measure implemented in the three parks is only body temperature testing before entrance. The effects and consequences of this measure should be reconsidered.

## 5. Conclusions

Research on COVID-19 and social distancing in parks by computer model simulations is a challenging task. Four important conclusions can be drawn from the current study. First, there is a high risk of COVID-19 spreading in urban parks. Adequate social distancing measures can only be maintained if the visitor numbers are strictly controlled. In different parks, the capacity is only 0.3% to 14.7% according to historical daily data. Second, congestions were found in the simulations in all park settings. The current visitor control, park layout, and site designs are not optimized for social distancing measures. The destinations in a 500 m radius to the most popular entrances, with a narrow long path inside, and with narrow openings are more dangerous targets. Third, a wide range of capacities were calculated and discussed for different critical infection periods or conditions. The current capacity can be easily adopted with future proof by linear increment. Finally, several policies and park adjustments were compared, and some were found to be effective, such as opening hour control, circular paths, wider openings, and AI warning boards at narrow entrances. It can be inferred that information provisions are more effective than space design adjustments and mandatory measures.

These results are aimed at providing a better understanding of parks under an epidemic background, which can improve public health safety. Moreover, this ABM simulation-based method can lead to bigger applications. Many public spaces are public health related. Testing contagions inside a park can be hard or unethical, while the observation of infections inside parks may be late already. The ABM simulation with no space simplification supplies a substitution for these unrealistic experiments. By entering different illness models, this method can be tested and verified in a variety of recreational spaces with many other contact-based illnesses. By comparing different parameters of different scenarios, better public health precautions can be selected. All these applications can provide important assistance when making important public health decisions. This not only extends mathematical and simulation methods in park visitor behavior studies under epidemical background but can also be applied in general research in all other public spaces.

ABM works more than as an experiment replacement. It can also be helpful in understanding the mechanism of public health-related issues. Previous studies are highly dependent on on-site survey and observations. These indirect indicators are helpful but limited. The ABM simulation provided a tool to study the feedback process of different parameters, which can improve the understanding of the interaction between the visitors and the settings. By checking the historical data of congested visitors in simulation, we find out that it is the lack of information that leads to their congestion. This feedback may one day greatly improve our knowledge of public health issues.

In addition to the application, the findings in the paper also revealed some properties of the public health problems in spaces as a complex system. In empirical tourism models, it is predicted that the narrow path can be the source of congestion. Meanwhile, based on the emerging knowledge, we can predict that microstructures will affect global property more. However, it is beyond estimation how greatly this microstructure property will enlarge the necessary safer space requirements for the visitors. Other properties, such as a marginal decreasing effect, local effect, and initial condition sensitive were also discovered in this paper. These findings based on ABM with no space simplification can be a great step in understanding the mechanisms and science of complex systems behind the public health issues.

Due to insufficient case reports of COVID-19 infections in parks and other open green spaces, the current study has not been verified yet. This is a general problem in all ABM simulation-based research. Big data and more repetitions can help to verify and improve some results. Behavior observations in parks in 2020 and 2021 will be a good supplement for categorizing visiting destinations and visitors, determining the percentage of visitors willing to keep distance. In western parks, the current results may be different due to varied layouts and usage patterns, which need more tests. The finite compute resources restrict the scale and complexity in the simulation, which may be resolved with future algorithms and computer science. The limited knowledge of complex system makes the theories and explanation developed in the paper require more tests and verification. However, this method based on ABM is still developing and has great potential.

## Figures and Tables

**Table 1:** Typical number of hourly park entrance visitors, employed in ABM for determining the number of visitors entering the park.

	West North Gate 2 of Bailuzhou Park		Xuanwu Gate of Xuanwu Lake Park		Main Parking lot of Sun Yat-sen Mausoleum	
Time	Weekday	Weekend	Weekday	Weekend	Weekday	Weekend
6–7	182	177	786	956	−	−
7–8	196	186	827	876	99	423
8–9	127	180	951	988	384	663
9–10	135	196	783	1246	654	1674
10–11	105	138	526	1457	423	1368
11–12	97	51	400	859	522	1533
12–13	106	104	373	712	435	1128
13–14	94	123	314	1159	360	792
14–15	72	132	365	1502	570	1260
15–16	133	190	576	1542	393	855
16–17	126	132	805	1511	417	870
17–18	90	161	868	1610	120	561
18–19	243	292	1954	2650	45	168
19–20	192	237	1086	2117	−	−

**Table 2:** Typical traveling time of park visitors, which was used to determine the length of stay for users in the park.

Period	Weekday			Weekend		
Place	Bailuzhou Park	Xuanwu Lake Park	Sun Yat-sen Mausoleum	Bailuzhou Park	Xuanwu Lake Park	Sun Yat-sen Mausoleum
<1 h	68%	73%	8%	56%	52%	7%
2 h	32%	10%	52%	42%	27%	58%
4 h	−	3%	33%	2%	16%	29%
>4 h	−	−	6%	−	5%	6%

**Table 3:** Typical composition of park visitor types, which was used to determine the types of behaviors for visitors.

Period	Weekday			Weekend		
Place	Bailuzhou Park	Xuanwu Lake Park	Sun Yat-sen Mausoleum	Bailuzhou Park	Xuanwu Lake Park	Sun Yat-sen Mausoleum
Exerciser	40%	29%	10%	34%	20%	5%
Leisure	24%	22%	7%	26%	24%	9%
Tourist	30%	40%	69%	28%	45%	83%
With children	3%	4%	-%	7%	7%	-%
Others	3%	4%	7%	4%	3%	3%

**Table 4:** The capacities of the three parks considering a critical period of 10 min to induce infection.

Park	Bailuzhou Park		Xuanwu Lake Park		Sun Yat-sen Mausoleum	
Time	Weekday	Weekend	Weekday	Weekend	Weekday	Weekend
Capacity of 10 min	380	370	709	652	128	93
Accessible area(hectare)	3.24	3.24	28.97	28.97	36.94	36.94
Percentage of trails area	64%	64%	64.3%	64.3%	52.9%	52.9%
Percentage of popular scenery area	7.4%	7.4%	9.8%	9.8%	37%	37%
Percentage of children facility area	3.9%	3.9%	2.7%	2.7%	0%	0%
Percentage of open space	24.7%	24.7%	23%	23%	9.7%	9.7%
Historical Visitor number	4267	5404	24,088	41,611	7964	18,288
Percentage of historical visiting number	8.9%	6.8%	2.9%	1.6%	1.3%	0.5%
Estimation by social distance area	2579	2579	23,065	23,065	29,201	29,201
Percentage of Estimation by social distance area	14.7%	14.3%	3%	2.8%	0.4%	0.3%
Capacity of 1 min	81	80	20	22	13	11
Capacity of 2 min	141	122	241	215	41	35
Capacity of 4 min	251	183	417	365	62	60
Capacity of 8 min	327	330	602	517	90	81
Capacity of 16 min	547	642	836	799	134	120
Capacity of 32 min	2016	1821	4109	3706	401	322
Capacity of 64 min	3129	2914	6226	5102	663	561

**Table 5:** Capacity of different visitor composition and correlation with site properties.

Visitor Composition	Capacity	Area per Person (m^2^)	Connection Width to Main Path (m)		
			Total	Minimum	Average
Weekday daily	380	85	561	2	13.8
Physical exerciser and social leisure	355	80.9	333	2	8.63
Average mixed	445	72	561	2	24.85
Physical exerciser	342	60.6	221	2	6.2
Tourists	235	10.3	156	2	6.9
Social leisure	300	26.6	112	2	14.3
Children entertainment	260	4.8	72	72	72
Correlation with capacity	1	0.85 *	0.88 *	−0.41	0.14

Connection width to main path: width of opening space at trailhead connecting to main path. * *p*-value less than 0.05.

## Data Availability

The data presented in this study are available on request from the corresponding author.

## Appendix A

Questionnaire on consumption behavior of parks in Nanjing

Dear friends,

Hello, this is an academic questionnaire, which aims to explore the consumption behavior of parks in Nanjing. We hope to have your support and assistance. There is no right or wrong answer in this questionnaire. Your answer is only for statistical analysis and academic research. This questionnaire is filled in anonymously, and your answers and personal information will be kept strictly confidential, please feel free to answer. We sincerely thank you for your support and assistance in this research.

1. How old are you?

☐Under 30 years old ☐30–60 years old ☐Over 60 years old

2. Where are you from?

☐Residents near the park ☐Nanjing natives ☐Visiting from city

3. What is your purpose?

☐Exercise ☐Leisure ☐Tourist ☐With children ☐Others

4. What is your planned visiting time in the park?

☐<1 h ☐2 h ☐4 h ☐>4 h

5. How much would you like to spend inside the park?

☐0 yuan ☐0–20 yuan ☐20–50 yuan ☐50–100 yuan ☐100 yuan and above

6. What mode of transportation did you use to arrive? (Multiple choices are allowed)

☐Bus (subway) ☐Self-driving ☐Taxi ☐Bicycle ☐Tourist bus ☐Electric car ☐Walking

7. How long did you spend on your way to the park?

A total of _ _ _ _ _ _ _ _ _ _ minutes.

## Appendix B

**Table A1:** Important citations of park capacity.

Year	Author	Topic and Achievement
1964	Wagar, J.A.	Developed the first formal exploration of the recreational carrying capacity concept
1998	Belnap, J.	The early study on visitor impact and visiting experience in Arches National Park
2015	Stankey, G.H.	The limits of acceptable change (LAC)
1990	Kuss, F.R.; Graefe, A.R.; Vaske, J.J.	Visitor Impact Management (VIM)
1997	U.S. Department of the Interior, National Park Service, Denver Service Center	Visitor Experience and Resource Protection (VERP)
2005	Andrés-Abellán, M.; Del Álamo, J.B.; Landette-Castillejos, T.; López-Serrano, F.R.; García-Morote, F.A.; Del Cerro-Barja, A.	The impact of trampling
2003	Garcia, C.; Servera, J.	Water demand
2009	Castley, J.G.; Hill, W.; Pickering, C.M.	Visitor use of protected areas
2011	Leung, Y.F.; Newburger, T.; Jones, M.; Kuhn, B.; Woiderski, B.	Visitor-created informal trails
2016	D’Antonio, A.; Monz, C.	Off-trail usage
2011	Tomczyk, A.M.; Ewertowski, M.	Trail degradation
2013	Mace, B.L.; Marquit, J.D.; Bates, S.C.	Mandatory alternative transportation systems
2014	Wolf, I.D.; Croft, D.B.	Impacts of tourism hotspots
2013	Pettebone, D.; Meldrum, B.; Leslie, C.; Lawson, S.R.; Newman, P.; Reigner, N.; Gibson, A.	Effects of crowding
2010	Pettebone, D.; Newman, P.; Lawson, S.R.	Effects of crowding
2016	Rasoolimanesh, S.M.; Jaafar, M.; Marzuki, A.; Mohamad, D.	Environmental characteristics
2002	Gössling, S.; Borgström Hansson, C.; Hörstmeier, O.; Saggel, S.	Ecological footprint
2015	Connell, J.; Page, S.J.; Meyer, D.	Seasonality
2018	Ghaderi, Z.; Abooali, G.; Henderson, J.	Community capacity
2009	Lai, P.H.; Sorice, M.G.; Nepal, S.K.; Cheng, C.K.	Social marketing
2012	Leung, X.Y.; Wang, F.; Wu, B.; Bai, B.; Stahura, K.A.; Xie, Z.	Overseas tourists
2018	Farías-Torbidoni, E.I.; Baric, D.; Anić, P.	Visitor sociodemographic
2020	Wu, Y.; Wang, L.; Fan, L.; Yang, M.; Zhang, Y.; Feng, Y.	Phone GPS
2020	Zhang, T.; Lian, Z.; Xu, Y.	Behavior observations, trail monitoring, animal habitats, soil, space syntax
2019	Barros, C.; Moya-Gómez, B.; García-Palomares, J.	Geotagged photographs
2019	Corbau, C.; Benedetto, G.; Congiatu, P.P.; Simeoni, U.; Carboni, D.	Web evaluation
2007	Heijman, T.L.J.; Van Der Bij, A.K.; De Vries, H.J.C.; Van Leent, E.J.M.; Thiesbrummel, H.F.J.; Fennema, H.S.A.	The park-carrying capacity during the epidemical period and contagiousness in natural landscapes
